# Movement-generated afference paired with transcranial magnetic stimulation: an associative stimulation paradigm

**DOI:** 10.1186/1743-0003-11-31

**Published:** 2014-03-05

**Authors:** Dylan J Edwards, Laura Dipietro, Asli Demirtas-Tatlidede, Ana H Medeiros, Gary W Thickbroom, Francis L Mastaglia, Hermano I Krebs, Alvaro Pascual-Leone

**Affiliations:** 1 Burke Medical Research Institute, White Plains, NY, USA; 2 Department of Neurology and Neuroscience, Weill Medical College, Cornell University, New York, USA; 3 Berenson-Allen Center for Non-Invasive Brain Stimulation, Harvard Medical School, Boston, MA, USA; 4 Centre for Neuromuscular and Neurological Disorders, University of Western Australia, Crawley, Australia; 5 Mechanical Engineering Department, Massachusetts Institute of Technology, Cambridge, MA, USA; 6 Department of Neurology, University of Maryland, School of Medicine, Baltimore, MD, USA; 7 Institut Guttmann, Universitat Autonoma de Barcelona, Barcelona, Spain; 8 Behavioral Neurology and Movement Disorders Unit, Department of Neurology, Istanbul University Istanbul Faculty of Medicine, Istanbul, Turkey

**Keywords:** Transcranial magnetic stimulation, Passive movement, Movement associative stimulation, Motor evoked potential

## Abstract

**Background:**

A peripheral nerve stimulus can enhance or suppress the evoked response to transcranial magnetic stimulation (TMS) depending on the latency of the preceding peripheral nerve stimulation (PNS) pulse. Similarly, somatosensory afference from the passively moving limb can transiently alter corticomotor excitability, in a phase-dependent manner. The repeated association of PNS with TMS is known to modulate corticomotor excitability; however, it is unknown whether repeated passive-movement associative stimulation (MAS) has similar effects.

**Methods:**

In a proof-of-principle study, using a cross-over design, seven healthy subjects received in separate sessions: (1) TMS (120% of the resting motor threshold-RMT, optimal site for Flexor Carpi Radialis) with muscle at rest; (2) TMS paired with cyclic passive movement during extension cyclic passive movement (400 pairs, 1 Hz), with the intervention order randomly assigned. Normality was tested using the Kolmogorov-Smirnov test, then compared to pre-intervention baseline using repeated measures ANOVA with a Dunnet multiple comparisons test.

**Results:**

MAS led to a progressive and significant decrease in the motor evoked potential (MEP) amplitude over the intervention (R^2^ = 0.6665, P < 0.0001), which was not evident with TMS alone (R^2^ = 0.0068, P = 0.641). Post-intervention excitability reduction, only present with MAS intervention, remained for 20min (0-10min = 68.2 ± 4.9%, P < 0.05; 10-20min = 73.3 ± 9.7%, P < 0.05).

**Conclusion:**

The association of somatosensory afference from the moving limb with TMS over primary motor cortex in healthy subjects can be used to modulate corticomotor excitability, and may have therapeutic implications.

## Background

A single transcutaneous electrical peripheral nerve stimulus (PNS) generates an afferent volley that can modify the excitability of sensorimotor cortex as assessed by a change in somatosensory evoked potential (SSEP) amplitude following the PNS [1], or motor evoked potential (MEP) amplitude following transcranial magnetic stimulation (TMS) [2], increasing or decreasing excitability according to latency of stimulus. Similarly, the afference generated by cyclic passive limb movement can modify cortical excitability in a transient and phase-specific manner, increasing during shortening and decreasing during lengthening [3-5]. Repetitive pairing of peripheral nerve stimulation with TMS (paired associative stimulation; PAS) can result in a change in MEP amplitude over time in a direction dependent on the inter-stimulus interval (ISI). An ISI of 10ms has an inhibitory effect, while 25ms has a facilitatory effect [6-8]. However, the repeated association of TMS with naturally generated afference such as that occurring during movement has not been reported in the literature. Based on the following factors, we propose that phase-specific cyclic passive movement when coupled with TMS repeatedly, may lead to a progressive modulatory effect: (1) the response to TMS depends on the state of the motor cortex at the time of stimulation [9], (2) that movement-generated afference can alter corticospinal output [3], (3) that consistent association of specific cortical afferent and efferent activity can modulate cortical excitability in animals [10] and humans [7,11]. Therefore, the aim of the present study was to investigate the effects of the TMS delivered during the inhibitory phase of cyclic passive movement on MEP amplitude.

## Methods

### Participants and study design

Seven right-handed healthy participants (2 female; age 21-46 years) volunteered after giving their written informed consent to the study approved by the Committee for Clinical Investigations at Beth Israel Deaconess Medical Center, Boston, MA and the MIT-COUHES (Committee on the Use of Humans as Experimental Subjects). Participants attended on two occasions separated by 1–7 days, in a within-subjects cross-over study design, with one of two interventions: (1) rTMS at rest and (2) rTMS delivered during passive limb movement. Each intervention (outlined below) was randomly assigned to the first or second visit.

### Subject position

Each subject was seated with a robotic device on the right side and secured at the hand, wrist, and above the elbow. Details of the robotic wrist device used have been previously reported [12]. The workstation was designed to fit the patient comfortably and allow approximately 20° of shoulder abduction and 30° of shoulder flexion. The forearm was positioned comfortably on a curved rest so that the ulnar styloid barely sat beyond the distal edge, while the hand was secured to the handle in a comfortable composite flexion grip with the least restrictive support provided. Humeral and forearm straps were supplied to maintain snug support.

### Passive movement

**Figure 1:**
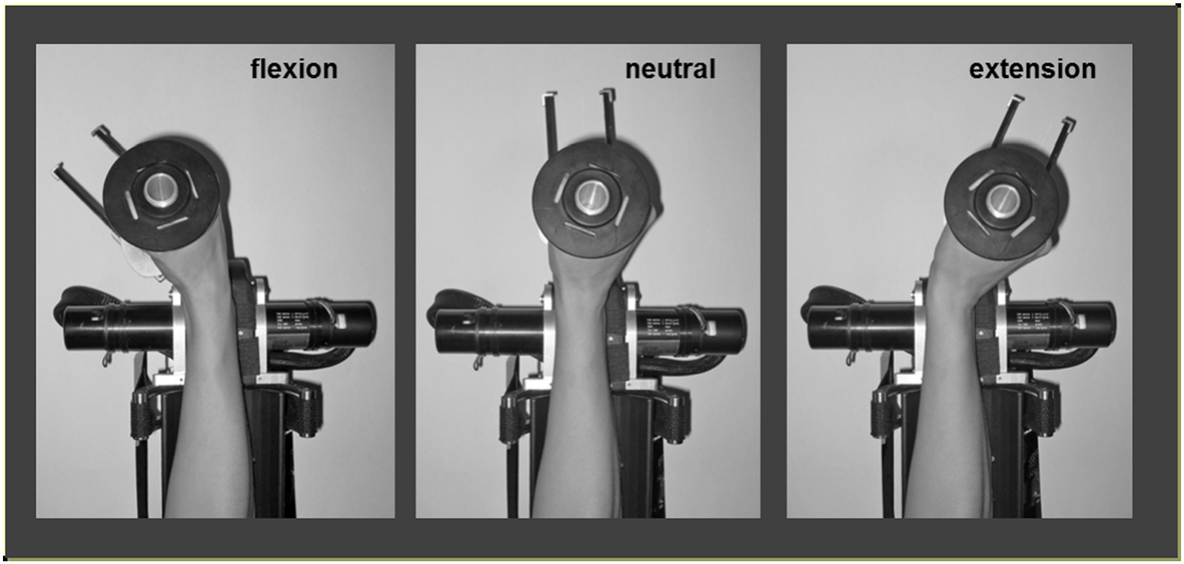
**Forearm and hand position in the robotic wrist device (upper view).** The forearm rests in a cushioned support in mid pronation-supination. The axis of rotation of the wrist is aligned with the axis of the device. The fingers were lightly fixed around the handle and supported with a velcro strap. The robotic device moves the wrist in a cyclic manner through flexion and extension with a smooth sinusoidal velocity profile. Movement is restricted to only the flexion-extension (sagittal) plane.

The weight of the wrist was supported by the robotic wrist device. During the movement intervention, the robot moved the wrist in a cyclic pattern of extension and flexion through a range of 90° at a rate of 1Hz. The velocity profile was bell-shaped, peaking as the wrist passed through mid-range flexion and extension (wrist neutral position, see Figure 1) [13,14].

### Electromyography

Surface electromyographic (EMG) activity was recorded from electrodes positioned over the muscle belly of the right flexor carpi radialis (FCR) muscle. EMG signals were amplified (×1000) and band-pass filtered between 10 and 1000 Hz, before being digitized at 2000 Hz for 100 ms following each stimulation, using a Powerlab 8/30 acquisition and analysis system (ADInstruments).

### Transcranial magnetic stimulation

A lycra cap was positioned over the head, with the vertex marked by measuring the mid-point intersection of the nasion-inion and inter-aural lines. Stimulus sites were then marked on the cap using the vertex as a reference point, in 1-cm steps in the coronal and sagittal planes, over the region of the left primary motor cortex. TMS was delivered using a MAGSTIM RAPID (Magstim Corporation) stimulator with a 5 cm diameter figure-of-eight coil, held tangential to the skull and aligned in the para-sagittal plane; the handle was rotated 45° laterally and the coil junction held over the optimal site of stimulation. The optimal site of stimulation for FCR, determined from initial exploration, was defined as the site with the largest MEPs for a given supra-threshold stimulus intensity, and used throughout the experiment. RMT (resting motor threshold) was determined with the wrist supported in the mid-range position, using four stimuli for each 1% increment of stimulator output intensity; it was defined as the minimum TMS intensity that evoked an MEP of at least 50μV amplitude in three of the four stimuli and then again confirmed with decreasing stimulus intensity. Ten single pulses at 120% RMT were delivered over the optimal site for FCR stimulation at baseline and each post-intervention time-point (using the baseline stimulus intensity). For the baseline measurement, a set of 10 stimuli was performed 10 minutes prior, and immediately before the intervention to ensure baseline stability. Post-intervention measurements occurred at 0, 5, 10, 15, 20, 25, and 30 minutes.

### Intervention

For both movement and resting interventions, 400 TMS pulses were delivered at 120% RMT and frequency of 1Hz (low frequency ≤1 Hz) [15,16]. At rest, TMS was delivered in the mid-range wrist position. During movement, TMS was triggered by the robotic device to coincide with the wrist passing through the position from flexion to extension, in the FCR muscle lengthening phase, at the same mid-range position (Figure 1, neutral). Throughout both interventions, subjects maintained muscle relaxation (monitored by real-time EMG).

### Data analysis

The peak-to-peak amplitude for each MEP was measured. The post-intervention data was grouped into three consecutive 10-minute blocks (0, 5, 10 min; 15, 20 min; 25, 30 min), and referred to as 0-10 min, 10-20 min and 20-30 min respectively. The mean MEP amplitude for each block was expressed as a percentage of mean pre-intervention amplitude. Group mean data at each time point were tested for normality using the Kolmogorov-Smirnov test, then compared to pre-intervention baseline using repeated measures ANOVA with a Dunnet multiple comparisons test.

Change in MEP amplitude over time and trends in mean MEP amplitude with time, during the intervention conditions (stimulation only or combined stimulation and movement), were tested using linear regression analysis. Group mean data for this calculation comprised the average of each successive 10 MEPs, giving 40 data points across the 400 repetitions, each expressed relative to the mean baseline amplitude. Post-intervention changes in MEP amplitude were established by comparison between each time-point post, and the pre-intervention baseline. Group data for pre-intervention and each time-point post-intervention were tested for normality using the Kolmogorov-Smirnov test and subsequent repeated measures ANOVA, with Tukey’s post-hoc comparisons.

To test the immediate effect of passive movement on MEP amplitude relative to the same stationary wrist position the mean of each successive 10 stimuli from repetition 1–30 was examined with respect to pre-intervention using repeated measures ANOVA, and the Dunnet multiple comparisons test. Data are presented as mean ± SEM. ‘Normalized’ data refers to expression as a percentage of pre-intervention baseline.

## Results

**Figure 2:**
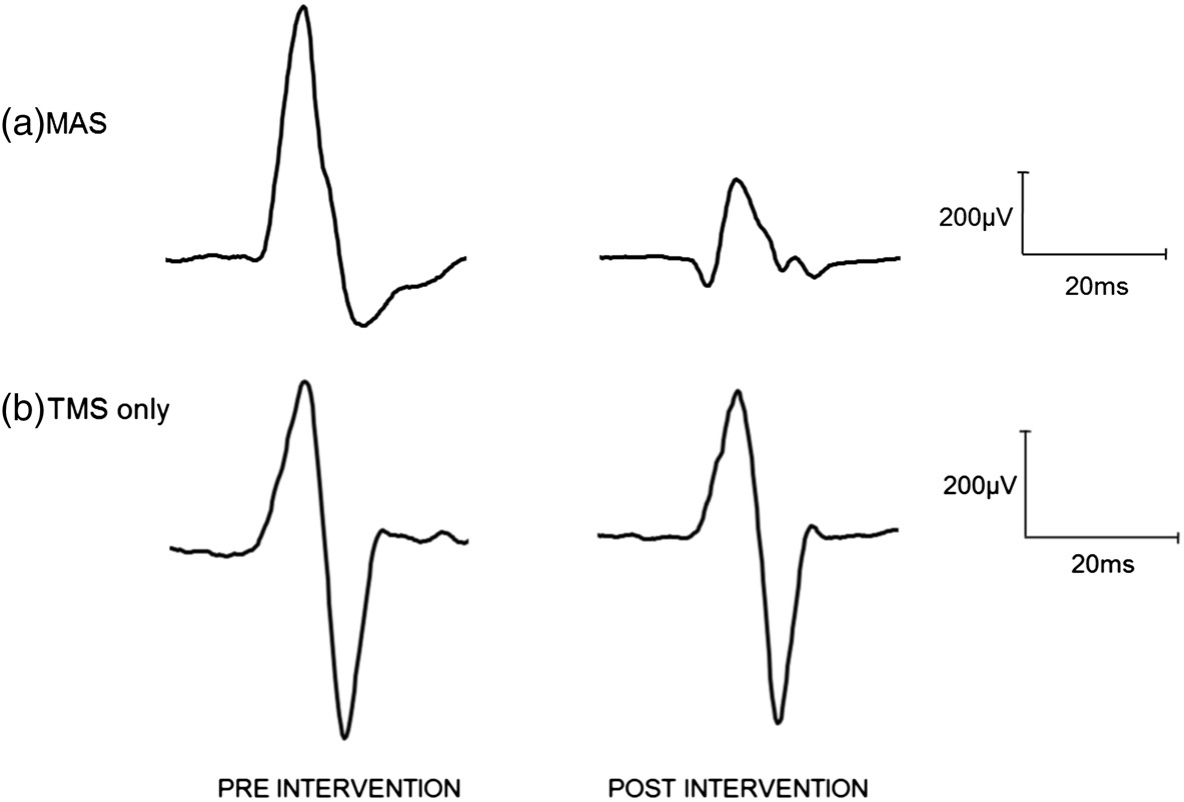
**Averaged waveforms from one subject at rest pre and post intervention. (a)** MAS intervention, and **(b)** TMS only intervention. Experimental sessions occurred on separate days. These results reveal that 400 repetitions of 1Hz TMS at rest may be insufficient to reduce MEP amplitude post intervention, yet when stimuli were timed to coincide with each wrist extension phase of passive movement, a reduction in MEP amplitude can be observed.

A marked reduction in the MEP amplitude was observed during the MAS intervention, but not for the TMS intervention alone. It can be seen in Figure 2, which shows representative sample resting MEP waveforms for one subject at baseline and post-intervention.

**Figure 3:**
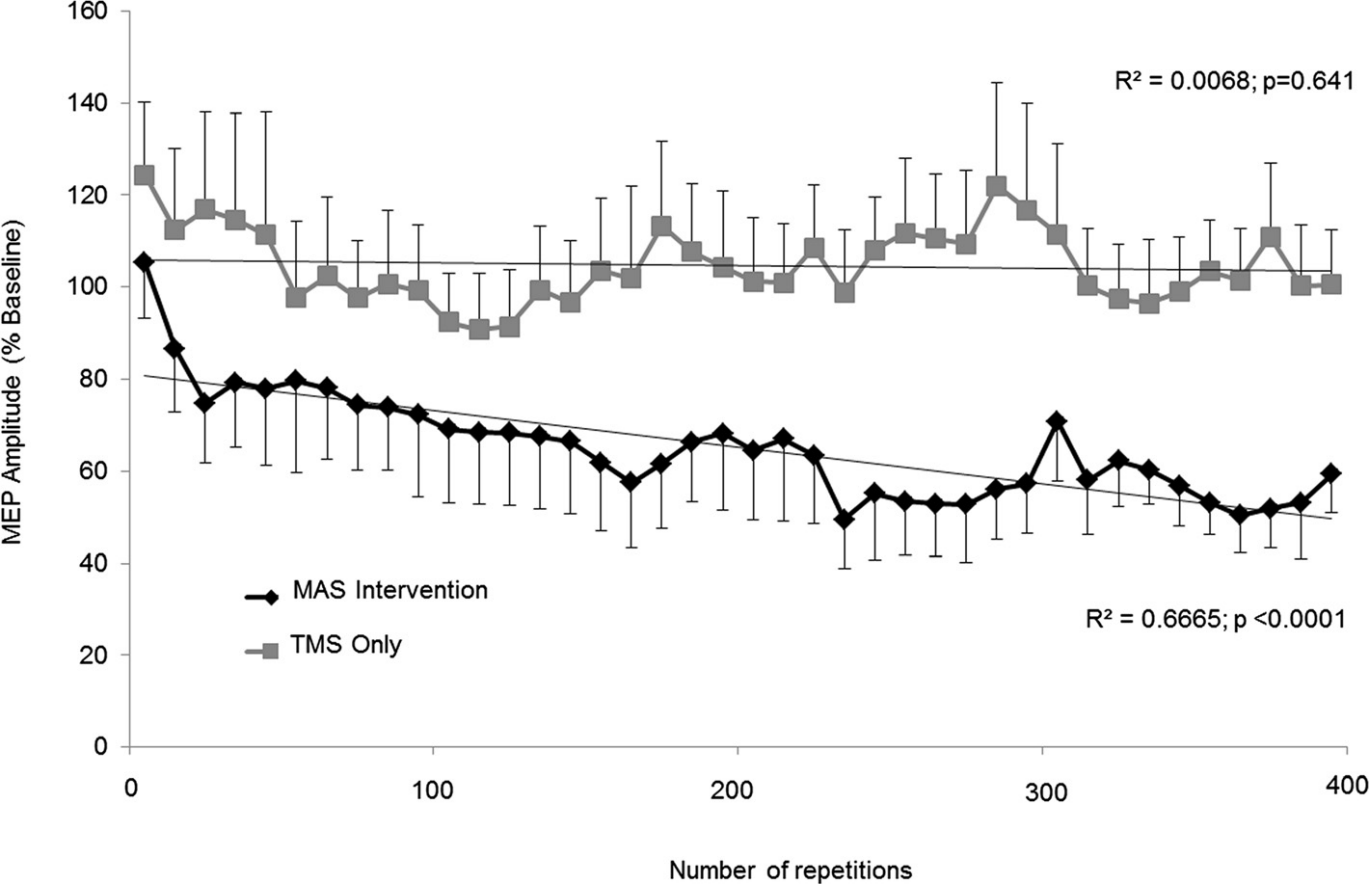
**MEP amplitude during intervention.** Mean (±SEM) MEP amplitude during intervention across subjects, normalized to pre-intervention at rest. MEPs were recorded from the FCR muscle at rest with the wrist in the mid-range position, or during passive wrist extension through the mid-range position. MEP amplitude progressively decreases during the MAS intervention, but not with the TMS intervention at rest.

During MAS intervention (rTMS during movement; Figure 3), MEP amplitude progressively decreased over the 400 repetitions (R^2^ = 0.6665, P < 0.0001). However, when the same stimulation was delivered with the wrist stationary in the mid-range position, no reduction in MEP amplitude was observed with 400 repetitive stimuli (R^2^ = 0.0068, P = 0.641). Additionally, movement-related reduction in MEP amplitude was present in some subjects at the onset of cyclic movement, yet the mean amplitude for the group was non significantly reduced until the third block of 10 repetitions [Rest = 0.46 ± 0.1. MAS intervention: 1st block = 0.47 ± 0.1 (105.3 ± 12%, P > 0.05); 2nd block = 0.36 ± 0.1 (88.7 ± 14%, P > 0.05); 3rd block = 0.29 ± 0.05 (74.8 ± 13%, P < 0.05)]. However, relative to pre-intervention baseline taken at rest (0.2Hz stimulation), the amplitude of the MEP response showed a tendency of increasing to the first 10 stimuli delivered during 1Hz resting intervention (124.4 ± 16% baseline; P > 0.05).

**Figure 4:**
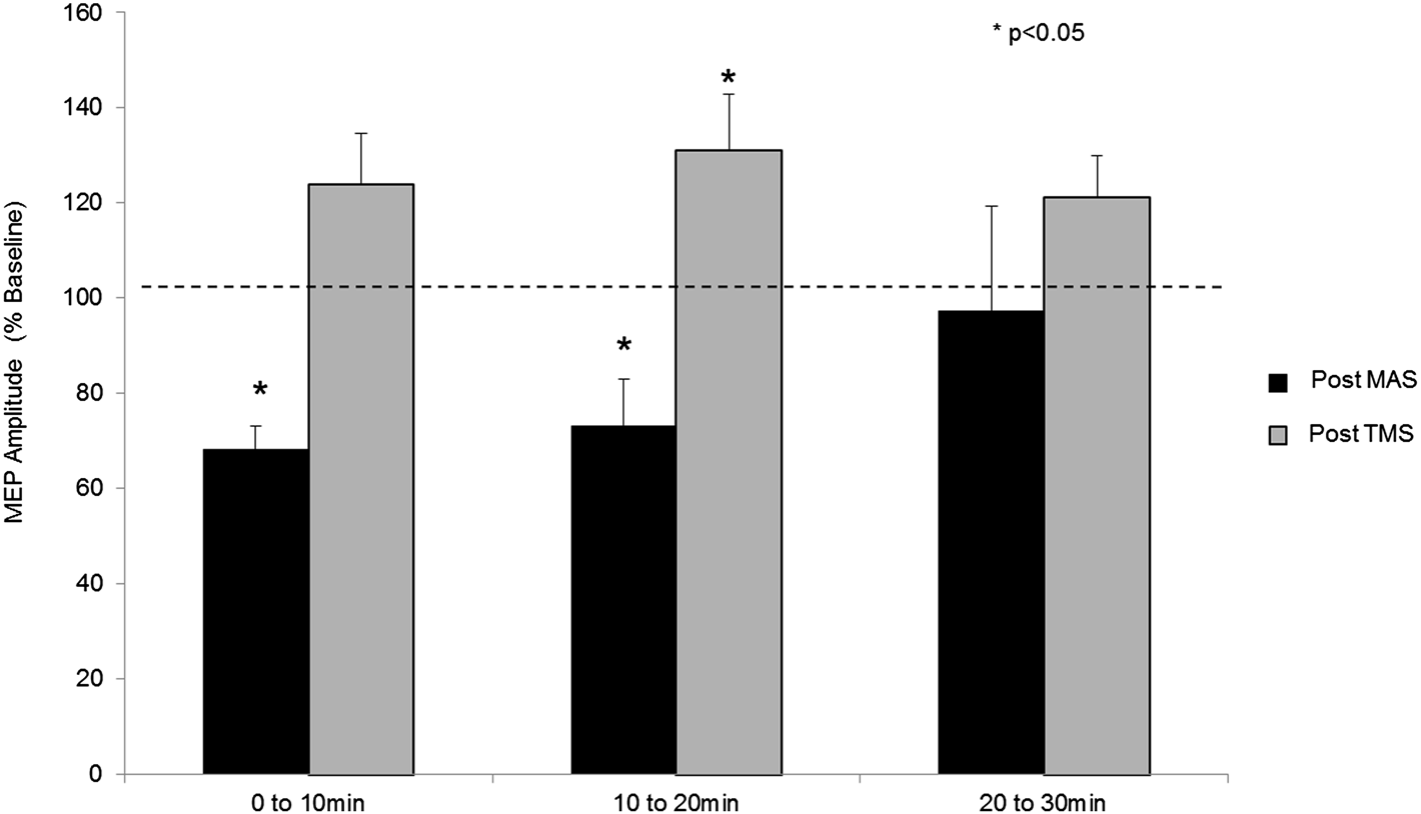
**MEP amplitude post-intervention.** Mean (±SEM) MEP amplitude across subjects at rest, for each post-intervention time period (normalized to pre-intervention amplitude). MEPs were significantly reduced for 20 min following MAS intervention, before returning to baseline by 30 min. MEPs were not reduced after stimulation only, and were significantly elevated at 10 to 20 min post-intervention.

Following movement intervention, MEP amplitude remained suppressed for 20 minutes, then returned to baseline between 20 and 30min post movement (0-10 min = 68.2 ± 4.9%, P < 0.05; 10-20 min = 73.3 ± 9.7%, P < 0.05; 20-30 min = 97.4 ± 22%; Figure 4). Following the TMS intervention only, however, a reduction in mean MEP amplitude was not observed. There was instead a mild increase that became significant between 10 and 20 minutes post-intervention and then resolved between 20 and 30 minutes (0-10 min = 124.0 ± 10.6%, P > 0.05; 10-20 min = 131.1 ± 11.8%, P < 0.05; 20-30 min = 121.3 ± 8.6%; Figure 4).

## Discussion

The present study provides evidence for the functional interaction of the repetitive coupling of phase-specific passive limb movement with TMS over primary motor cortex, which progressively reduced human corticomotor excitability and was sustained for 20 minutes following the combined intervention, yet no such reduction was observed during or following intervention with the stimulation only. These results show for the first time that naturally occurring afference generated by the passively moving limb can be harnessed to interact with corticomotor activity from TMS in an associative manner, such that when performed repetitively, and has a short-term neuromodulatory effect.

In order to favor an excitability reduction modulatory effect in the present study, we selected both rTMS stimulation rate and passive movement rate accordingly. In this regard, an effect of passive movement was demonstrated across the first 30 repetitions, that may be stronger if delivered at a lower stimulation frequency – i.e. 0.2Hz shown in one subject and consistent with our previous work [17]. That is, in the early cycles of movement, reduced amplitude MEPs were observed, stronger with slower stimulation rate, yet not present in the absence of movement (during 30 reps of 1Hz rTMS only). Movement caused an immediate reduction in MEP amplitude, with a further progressive and significant reduction when repeated for 400 cycles. This led to an effect of reduced MEP amplitude that persisted following the intervention period, and then returned to baseline within 30 minutes. Stimuli delivered at the end of range of muscle lengthening resulted in no change in MEP amplitude during intervention (immediately or progressively) or after the intervention, supporting previous literature that excitability changes are phase specific [5], and that passive movement alone at this rate does not have cumulative effects on MEP amplitude [17].

In this study we compared the effects of rTMS-movement paired stimulation, with the effects of the same low frequency (1Hz) stimulation alone by delivering 400 stimuli at 120% of the RMT at rest and found no suppression in the MEP size following this intervention. Previous studies of 1Hz stimulation over human primary motor cortex at rest show a short-term reduction in CM excitability [18,19]. However, this effect appears to be related to stimulation intensity as well as the number of applied stimuli [20]. For in vitro models of long-term depression (LTD), 600–900 pulses are needed for a consistent and sustained effect [21,22], whereas 800–1600 pulses are typically required in humans since the effects can be quite variable across subjects [23]. We specifically used 1Hz rTMS, but with insufficient repetitions to exert CM excitability reduction in the absence of movement. We showed that stimulation alone over 400 repetitions did not reduce MEP amplitude, but rather a trend of increased excitability was present, although the significance of an increase between 10 and 20 minutes post-intervention is difficult to explain.

Of note during the intervention, was the different onset and trajectory of MEP amplitude reduction between individuals. The variance in intervention effect time-course has been observed previously with other repetitive non-invasive stimulation protocols (iTMS, [24]; SAS, [11]). We suspect that while the dose of stimulation (e.g. threshold adjusted stimulation intensity) might be consistent across subjects for neuromodulation paradigms such as this, the individual response time-course will be different (for a host of reasons, including brain state). Until real time individual dose–response is sufficiently considered, we might expect variance in the lasting excitability changes. This remains to be further explored in the protocol reported here, as well as other neuromodulation protocols.

It is possible that the high stimulus intensity results in a net facilitatory effect similar to paired pulse techniques that switch from inhibitory to facilitatory MEP response, with increases in intensity above motor threshold. Furthermore, the number of repetitions at this intensity may be too small to finally result in a decreased excitability. The MEP amplitude fluctuates during low-frequency rTMS protocols ultimately leading to sustained inhibition, including changes in cortical facilitatory and inhibitory network activity, and intervening homeostatic mechanisms. Change in MEP amplitude is not readily observable in most protocols since the stimulus intensity is sub-threshold. Increased MEP amplitude may be a normal phenomenon in the early phase of 1Hz inhibitory protocols, or a result of the relatively high-intensity stimulation used in the current protocol. Increased stimulus intensity from sub-threshold to supra-threshold (80–115% RMT) has previously been shown to augment the post-intervention excitability change (a greater reduction in MEP amplitude), although it is not known if this occurs early in the stimulation period, or if it occurs at intensities above 115% such as in the present study [23]. While both sub- and supra-threshold stimulation produce changes in the MEP, the effects tend to exhibit a more reliable and robust pattern with prolonged number of stimuli [19,25] at supra-threshold intensities [15,20,26,27]. A recent review investigating the effects of low frequency stimulation on MEP size reported a reduction in 13 of the 19 identified studies and pointed to clear intensity-related effects since insignificant reductions were evident at relatively low sub-threshold intensities [28]. In view of the current literature then, our findings of no reduction in MEP amplitude with the short intervention duration and high stimulus intensity are not surprising.

Peripheral limb movement forms a large basis for motor rehabilitation, and repetitive passive movement can lead to a temporary reduction in spasticity and orthopedic benefit [29-31]. In fact, cyclic passive movement can strongly reduce CM excitability during muscle lengthening [3,17,32]. This phenomenon may be dependent on the frequency of stimulation [15] and movement rate [33]. The mechanism of action is incompletely understood, however is thought to result from effects of muscle spindle afference at spinal and cortical level [4]. The strong effects of movement-related afference are transient, and may be completely reversed within 100ms of movement cessation [17]. This suggests that passive movement alone may be not result in any sustained change in excitability, which would be consistent with previous results [4]. The implication for the findings of the present study is that passive movement during the muscle lengthening phase of movement might have therapeutic application in disorders of hypertonicity. However, more broadly, these findings suggest that the ability of rTMS protocols to modulate cortical excitability may be influenced by interventions (such as passive movement) aimed at controlling cortical excitability.

The peripheral afference leads to a cumulative and lasting effect that could occur at spinal and/or supraspinal levels. Furthermore, the inhibitory phase of cyclic passive movement need not necessarily be complementary to an inhibitory rTMS protocol, and it may cancel the inhibitory effect, remain unchanged, or lead to an excitatory effect. For example, we know that decreased afferent activity (associated with increased MEP amplitude) also appears to increase the efficacy of low frequency rTMS, as shown during a study of peripheral nerve block [34]. Moreover, the mechanism of our observed effect cannot be elucidated from the current protocol, yet rTMS and passive movement have separately been shown to alter both spinal [35] and cortical excitability [4]. The circumstances under which cortical and/or spinal excitability changes occur are influenced by the nature of the neuromodulatory protocol, where paired associative stimulation for example can change cortical but not spinal excitability [7]. In the present study, both spinal and supraspinal excitability changes could contribute to our findings, however this remains to be determined.

Our findings raise the question of whether this type of associative paradigm could be used to increase cortical excitability. In principle, the association of the facilitatory phase of movement with TMS repeatedly, may increase cortical excitability over time consistent with long-term potentiation, as currently is well demonstrated with PAS [7,36], yet this remains to be proven experimentally.

## Conclusion

The association of somatosensory afference from the passively moving limb with TMS over primary motor cortex in healthy human subjects can be used to modulate corticomotor excitability, outlasting the intervention period. Parameters of TMS stimulation (rate, intensity, duration, pulse-shape) and movement (phase, rate, number of repetitions) require further investigation for the development of the optimal effect. It remains to be determined whether our findings could be applied to the treatment of disorders involving increased muscle tone such as spasticity and dystonia.

## Abbreviations

TMS: Transcranial magnetic stimulation; PNS: Peripheral nerve stimulation; MAS: Movement associative stimulation; RMT: Resting motor threshold; MEP: Motor evoked potential; SSEP: Somatosensory evoked potential; ISI: Inter-stimulus interval; EMG: Surface electromyographic; FCR: Flexor carpi radialis; rTMS: Repetitive transcranial magnetic stimulation; CM: Corticomotor.

## Competing interests

The authors declare that they have no competing interests.

## Authors’ contributions

DJE: experimental work, concept, study design and manuscript preparation. APL: concept, data interpretation. HIK: concept, experimental work, technical set-up, data analysis. LDP: data analysis, experimental work. GWT: data analysis/interpretation, manuscript preparation. ADT: data collection, data interpretation. AHM: data interpretation, manuscript development and preparation. FLM: concept, data interpretation and manuscript preparation. All authors wrote, read and approved the final manuscript.
